# Assessment of wild leafy vegetables traditionally consumed by the ethnic communities of Manipur, northeast India

**DOI:** 10.1186/s13002-016-0080-4

**Published:** 2016-01-29

**Authors:** Biseshwori Thongam, Surjata Konsam, Arun Kumar Handique

**Affiliations:** Plant Systematics and Conservation Laboratory (PSCL), Institute of Bioresources and Sustainable Development (IBSD), Takyelpat, Imphal, 795001 Manipur India; Department of Biotechnology, Gauhati University, Guwahati, 781014 India

**Keywords:** Wild edible plants, Local market, Integrated assessment, Sustainability, Food and nutritional security, Manipur

## Abstract

**Background:**

The NE region of India falls in the global hotspot of biodiversity. Wild edible plants (WEPs) are widely consumed in the daily diet of the local people. WEPs are critical for the sustenance of ethnic communities and also as a source of income. However, WEPs received a little attention in research activities, economic development, biodiversity conservation and sustainable management. Many are largely ignored and remained unexplored. With a view of reducing the gap in traditional knowledge and tapping the hidden potential resources for proper utilization, exploitation, and sustainable management of WEPs are crucial.

**Methods:**

Surveys were conducted at 20 major markets in all districts of Manipur throughout different seasons from August 2012 to March 2014. A total of 154 avid plant collectors and sellers were interviewed using semi-structured questionnaire, formal, informal and extensive interactions to gather detailed information about these species. An integrated assessment of 68 wild leafy vegetables was also carried out to prioritize them for proper exploitation, conservation, and sustainable management.

**Results:**

A total of 68 wild edible vegetables belonging to 42 families were documented which are being used by indigenous communities for nutritive and therapeutic purposes. Of these species, 54 are perennial (79 %) while others are annual (19 %). Herbaceous plants make up the highest proportion of edible plants. Leaves are dominant edible part followed by shoot and stem, and most are consumed through cooked food. Further, 57 species (84 %) are commonly available, and 11 (16 %) are rare. According to integrated assessment, 2 species have highest integrated value, 26 species have high value, 31 species have general value and 9 species are of low value. The majority of the species have a high or general value.

**Conclusion:**

Manipur has rich wild vegetable resources. However, many of them are seldom collected or cultivated given their importance in sustaining and diversifying diet. A comprehensive assessment indicated that majority of these plants have high value. Priority species require further research into their nutritional components to understand the potential as a source of future food and nutritional security. They should be promoted for integration into the agricultural system and income generation for local sustenance.

**Electronic supplementary material:**

The online version of this article (doi:10.1186/s13002-016-0080-4) contains supplementary material, which is available to authorized users.

## Background

At present, about 90 % of global food production comes from less than 30 species and more than 85–90 % of total caloric intake obtained from 12 domesticated species [1]. This situation may create an intense biotic and abiotic pressure to the modern agriculture in future. The majority of the edible plants are neglected which grow naturally in the wild and do not have to be tended before producing edible parts [2]. Such edible wild plants can significantly increase sustainability by reducing the risk of over-dependence on a limited number of crops.

The use of wild plants as food is an integral part of the culture and tradition of many indigenous communities around the world. A large section of the rural population meets their nutritional requirement through unconventional means, by consuming various wild plants and animal resources [3]. Millions of people, mostly in developing countries, derive a substantial part of their subsistence and income from wild plant products [4]. WEPs constitute an essential component in the variation of diet and bring household food security of many ethnic communities.

WEPs provide staple food for indigenous people and serve as complementary food for non-indigenous people and offer an alternative source of income [5–7]. They are an important source of nutrient, vitamin and mineral supplements for indigenous population [8, 9] and hence, reduce the vulnerability of local communities to food insecurity and thereby act as a buffer for food shortage during the emergency [10, 11]. Several researchers also demonstrated that many WEPs have nutritional or therapeutic value due to the presence of biologically active compounds, and therefore, can be considered as food-medicine and quality food [11, 12]. Many traditional leafy vegetables have higher nutritional values than several known common cultivated plants [13, 14].WEPs have substantial potential to increase the sustainability of agriculture through the reduction in multi-agricultural input. They can also be used for the development of new crops through domestication and benefit modern agriculture by providing plant breeders with a broad pool of potentially useful genetic resources for crop improvement [15, 16]. The genes for higher productivity and distinctive quality traits may be hidden in this gene pool.

Research on wild food plants is still active even in the present day. Such research is carried out in many countries and continents [17–20]. In the Indian subcontinent, 9500 wild plants are used for food, medicine, fodder, fiber, fuel, essence, cultural and other purposes by over a 53 million tribes belonging to 550 different communities [21]. Ethnobotanical studies on wild food plants associated with tribal communities of central India, Tamil Nadu, Maharashtra, Northeastern India, etc. [21–24] are reported from India. The tribal communities of the Himalayan region of India use over 195 wild edible species [25]. Wild food plants and vegetables being sold in the local markets of South Korea, Croatia, and Turkey [26–28] have also been reported. The local market provides much information on the ethnobotanical process of plant-people interaction and relationships. It represents an intensified interaction between people of different socio-economic groups and specific plants as well [29].

In spite of their immense importance as a valuable food source, WEPs remain widely unknown. Many of the wild food plants are restricted to certain areas or communities. Given the rapid decline of traditional knowledge about WEPs and increased reliance on processed food, documentation and evaluation of the traditional knowledge related to the diversity, usage, and status of WEPs are crucial. Some studies on ethnomedicinal plants have been conducted in Manipur; however, there is limited information on wild vegetables despite its diverse uses. Moreover, information on the nutritional values of most of the WEPs of Manipur is not available. Research and development activities to tap these assets for economic development and sustainability have also remained at the bottom. Many more wild species believed to be edible are yet undocumented. The rich biodiversity of wild plants will be useful in screening newer source of vegetables for present and future need. Inventory of wild food resources, ethnobotanical information on their diversity, usage, status, etc. coupled with nutritional evaluation can establish native species as an alternative to achieve food and nutritional security.

Our objective is to document and assess the diverse wild vegetable resources sold at the local markets of Manipur throughout different seasons. It also aims to provide a systematic way for prioritizing high-quality species through an integrated assessment. It will be utilized further for evaluation of nutritional components of priority species, their integration into the agricultural system and sustainable conservation and management.

## Materials and methods

### Study area

**Fig. 1:**
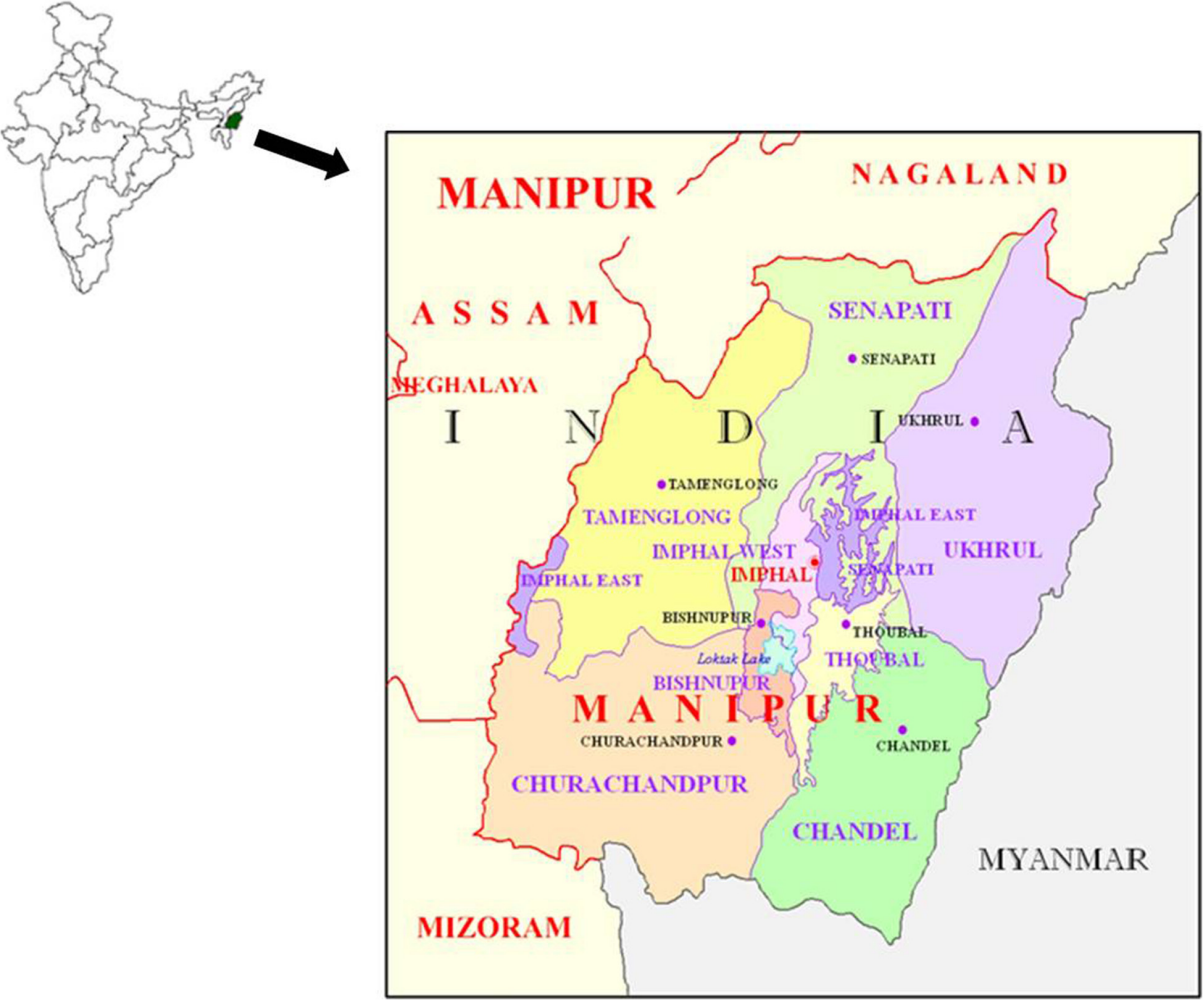
Location map of study site in Manipur, Northeast India

This study was carried out in Manipur, one of the seven states of Northeast India that forms an integral part of the Indo-Burma biodiversity hotspot. The Manipur state (23°27’ to 25°41’ N latitude and between 93°61’ and 94°48’ E longitude) comprises an area of 22, 327 km^2^ and administratively divided into 9 districts, of which 4 are valley (viz. Imphal East, Imphal West, Thoubal and Bishnupur) and the rest 5 are hill districts (Chandel, Churachandpur, Senapati, Ukhrul and Tamenglong (Fig. 1). The state is rich in both cultural and biological diversity, having populated by diverse ethnic, linguistic and religious groups including many indigenous tribes. Racially, Manipuri people are unique and have features similar to Southeast Asian. The state has four major ethnic communities - Meitei (Hindu), Naga and Kuki (Tribal communities) and Pangal (Muslim). The Meiteis are the dominant non-tribal community constituting 92 % of the valley area along with the Pangal (minority group), and the five hill districts are inhabited by about 34 ethnic tribes representing 30 % of the state population. They practice distinct culture and tradition and have different socio-economic features. Agriculture is the single largest occupation in Manipur and the mainstay of the state’s economy. The trade of wild vegetables provides an alternative source of income and is mainly done by women. Forests account for 67 % of the total land area of this state. The tribal communities collect a large variety of edible and other useful plants from the forest and surrounding wasteland. They also sell a large variety of such plants in the local market. The famous “Ima Keithel” (meaning “Mother’s market”) of Manipur which sells vegetables and other household items are exclusively run and controlled by women signifying their role in the society both socio-cultural and economically.

A total of 20 major markets were chosen for this study as they form the primary source of supply for wild edible plants in the state –viz 1. Imphal East - Lamlong bazaar and Chingmeirong bazaar 2. Imphal West - Khwairamban keithel and Singjamei bazaar 3. Thoubal District - Thoubal bazaar and Kakching bazaar 4. Bishenpur District - Nambol bazaar, Bishenpur bazaar, Ningthoukhong bazaar and Moirang bazaar 5. Chandel District - Chandel main market and Pallel bazaar 6. Churachandpur District - New Lamka bazaar and Tuibong bazaar 7. Senapati District - Kangpokpi bazaar and Senapati bazaar 8. Ukhrul District - Yaingangpokpi bazaar and Ukhrul main market 9. Tamenglong District - Noney bazaar and Tamenglong bazaar.

## Methods

### Survey and data collection

The methods employed in this study were designed for collecting baseline information on the diversity and usage of wild vegetable resources locally used by people of Manipur. Before conducting the survey, prior information consent was obtained from the interviewee by explaining the aim of the study. Participants in the study were selected by purposive sampling method. The criterion was to understand and obtain maximum possible information on edibility, medicinal, dietary preference, cultural association and market of wild vegetables from various user communities to come to a generalized inference on WEPs.

Markets were surveyed to assess the presence and abundance of wild edible plants. Detailed studies were conducted at 20 major markets in all districts of Manipur from August 2012 to March 2014 in different season. Each of this market was examined twice in every season between 6.00 and 9.00 am and 2–5 pm. A total of 154 semi-structured interviews were carried out with 130 female and 24 male in the age group of 30–77 years for the collection of data. Whenever necessary, translators were used while collecting data as the participants belong to different ethnic communities. However, a majority of them know Manipuri, the state language. Detailed information was gathered using formal, informal and extensive interactions with the wild plant vendor and those involved in the collection and marketing of WEPs following the methods of Upetry et al. [18] and Jain et al. [30]. The inquiries comprise their local names, sources, life forms, growth habit, availability period, edible part, mode of consumption, availability status, distribution pattern, and mode of propagation (Table 1). The collected specimens were identified with the help of experts, relevant literature and Flora [31–34]. The plant nomenclature and author abbreviations follow The Plant List [35]. The specimens were deposited in the Herbarium of Plant Systematics and Conservation Laboratory, Institute of Bioresources and Sustainable Development, Imphal, Manipur.

**Table 1:** Names, Life forms, Growth habit, Edible parts, Mode of utilization, Availability period and Availability status

Local names (Voucher no.)	Scientific names	Family	Life forms	Growth habit	Edible part(s)	Mode of utilization or preparation	Local availability period	Local availability status (in its season)
Chuchurangmei IBSD/WEP 001	*Sesbania sesban* (L.) Merr	Leguminosae	Annual	Shrub	Fruit, leaf	Raw leaves added to singju, fruit cooked eaten as eromba	August-September	Common
Kolamni IBSD/WEP 002	*Ipomoea aquatica* Forssk	Convolvulaceae	Perennial	Herb	Stem	Cooked eaten as vegetable	Year round	Common
Komprek IBSD/WEP 003	*Oenanthe javanica (*Blume) DC.	Apiaceae	Perennial	Herb	Leaf, stem	Eaten raw in singju or cooked as mixed vegetable	Year round	Common
Sinjupaal IBSD/WEP 004	*Alocasia cucullata* (Lour.) G. Don	Araceae	Perennial	Herb	Corm	Eaten raw as singju or cooked with potato and dry fish	Year round	Common
YelangIBSD/WEP 005	*Polygonum barbatum* L.	Polygonaceae	Annual	Herb	Leaf	Cooked eaten as vegetable	January-March	Common
Kakthum IBSD/WEP 006	*Eleocharis dulcis* (Burm.f.) Trin. ex Hensch	Cyperaceae	Perennial	Herb	Root	Eaten raw or steam as snack, also cooked eaten as eromba	November-December	Common
Ching yensil IBSD/WEP 007	*Antidesma diandrum* (Roxb.) B*.*Heyne. ex Roth	Euphorbiaceae	Perennial	Small tree	Leaf	Cooked eaten as eromba or with potato and dry fish	April -July	Common
KengoiIBSD/WEP 008	*Lysimachia ovovata* Buch.-Ham. ex Wall	Primulaceae	Perennial	Herb	Whole part	Cooked eaten as eromba, or with potatoes and dry fish	Winter	Common
PerukIBSD/WEP 009	*Centella asiatica (*L.) Urb.	Apiaceae	Perennial	Creeper	Whole plant	Boil eaten (champhut), or with potato and smashed with chilli, fermented fish (kangsu)	Year round	Common
Thamou IBSD/WEP 010	*Nelumbo nucifera* Gaertn.	Nelumbonaceae	Perennial	Rooted hydrophyte	Leaf, root	Eaten raw snack or singju; cooked with honey	June- October	Common
TharoIBSD/WEP 011	*Nymphaea nouchali* Burm.f.	Nymphaeaceae	Perennial	Rooted hydrophyte	Stem, tuber	Eaten raw - singju; boiled tuber eaten as snack	July- October	Common
Thangjing IBSD/WEP 012	*Euryale ferox* Salisb.	Nymphaeaceae	Annual	Rooted herb	Seed, stem	Eaten raw mixing with chilli and fermented fish or singju, added to cooked dish	June-September	Common
Esing ekaithabi IBSD/WEP 013	*Neptunia oleracea* Lour.	Leguminosae	Annual	Herb	Shoot	Raw as singju, cooked with other vegetables	Rainy season	Common
Koukha IBSD/WEP 014	*Sagittaria sagittifolia* L.	Alismataceae	Perennial	Herb	Tuber	Cooked eaten as eromba or fried with gram flour	November-January	Common
Yendang IBSD/WEP 015	*Cycas pectinata* Buch.-Ham	Cycadaceae	Perennial	Shrub	Leaf, shoot	Raw as singju or cooked eaten as eromba	June-September	Uncommon
Monsaobi IBSD/WEP 016	*Chenopodium album* L.	Amaranthaceae	Annual	Herb	Leaf	Cooked eaten with other vegetables	June-September	Common
Kanghumaan IBSD/WEP 017	*Meriandra bengalensis (*Roxb.) Benth.	Lamiaceae	Perennial	Shrub	Inflorescence, leaf	Added raw as dressing in eromba or singju	November- March	Common
Tekta IBSD/WEP 018	*Pogostemon purpurascens* Dalzell	Lamiaceae	Annual	Shrub	Leaf	Added as spices	September-October	Uncommon
Yerum keirum BSD/WEP 019	*Stellaria media* (L.) Vill.	Caryophyllaceae	Annual	Herb	Whole plant	Cooked as vegetable	Winter season	Common
Toninkhok IBSD/WEP 020	*Houttuynia cordata* Thunb.	Saururaceae	Perennial	Herb	Whole plant	Use as spice or accessory additives	Year round	Common
Loklei IBSD/WEP 021	*Hedychium coronarium* J. Koenig	Zingiberaceae	Perennial	Herb	Rhizome	Boiled eaten as eromba	April-May	Common
Pullei IBSD/WEP 022	*Alpinia nigra (*Gaertn.) Burtt	Zingiberaceae	Perennial	Herb	Rhizome	Boiled eaten as eromba	April- July	Common
Namra IBSD/WEP 023	*Amomum aromaticum* Roxb.	Zingiberaceae	Perennial	Herb	Stem	Boiled eaten as eromba	April- September	Common
Yaipal IBSD/WEP 024	*Curcuma angustifolia* Roxb.	Zingiberaceae	Perennial	Herb	Inflorescence	Boiled eaten as eromba, cooked, as well as fry eaten	April- May	Common
Sarei mapan IBSD/WEP 025	*Amomum* sp.	Zingiberaceae	Perennial	Herb	Inflorescence	Cooked eaten as eromba and as mixed vegetable fry	February-May	Uncommon
Esing kambong IBSD/WEP 026	*Zizania latifolia* (Griseb.) Turcz. ex Stapf	Poaceae	Perennial	Herb	Culms	Raw-snack, roast, cook as vegetables, cook with milk honey and black rice	September- November	Uncommon
Chantruk mana IBSD/WEP 027	*Cardamine hirsuta* L.	Brassicaceae	Annual	Herb	Leaf	Added raw in singju, additives in cooked curry	November-February	Common
*Huikhong/mansam IBSD/WEP 028	*Viola pilosa* Blume	Violaceae	Perennial	Creeper	Whole plant	Added raw in singju, cooked eaten as eromba and kangsu	Year round; except winter	Uncommon
Phunil IBSD/WEP 029	*Gnaphalium indicum* L.	Asteraceae	Annual	Herb	Whole part	Eaten raw as dressing, cooked eaten as vegetable	October-December	Common
Kongouyen IBSD/WEP 030	*Cissus javanica* DC	Vitaceae	Perennial	Climber	Leaf, stem	Cooked by boiling with potatoes and dry fish	Rainy season	Common
Heibi mana IBSD/WEP 031	*Vangueria spinosa* (Roxb. ex Link) Roxb.	Rubiaceae	Perennial	Tree	Leaf	Added raw in singju	Year round	Common
Lam khamen IBSD/WEP 032	*Solanum torvum* Sw.	Solanaceae	Perennial	Shrub	Fruit	Cooked eaten as vegetable	July-September	Common
Nongmangkha IBSD/WEP 033	*Phlogacanthus thyrsiformis* (Roxb. ex Hardw.) Mabb	Acanthaceae	Perennial	Shrub	Inflorescence	Eaten raw with chutney, cooked with other vegetable	December-March	Common
Oosingsha mapaan IBSD/WEP 034	*Litsea cubeba* (Lour.) Pers.	Lauraceae	Perennial	Tree	Inflorescence, fruit	Eaten raw with chutney, cooked as eromba,	November-April	Common
Chigonglei angouba IBSD/WEP 035	*Leucaena leucocephala* (Lam.) de Wit	Leguminosae	Perennial	Tree	Fruit	Eaten raw as singju, cooked as eromba	October- December	Common
Oothum IBSD/WEP 036	*Wendlandia paniculata* (Roxb.) DC	Rubiaceae	Perennial	Tree	Tender leaf	Cooked eaten as eromba with black pea or making chutney	March-April	Common
Mukthrubi IBSD/WEP 037	*Zanthoxylum acanthopodium* DC.	Rutaceae	Perennial	Tree	Leaf, inflorescence	Eaten raw with chili and fermented fish chutney or additives in snail curry	Year Round	Common
Naoseknambi IBSD/WEP 038	*Zanthoxylum* sp.	Rutaceae	Perennial	Tree	Leaf	Raw-singju, added in meat curry, cooked eaten as kangsoi	April-June	Common
Awaphadigom IBSD/WEP 039	*Eryngium foetidum* L.	Apiaceae	Perennial	Herb	Leaf	Added as spice in all cooked dish; especially in meat curry	Year round	Common
Heiba mana IBSD/WEP 040	*Exbucklandia populnea* (R.Br.ex Griff.)R.W.Br.	Hamamelidaceae	Perennial	Tree	Leaf, tender shoot	Eaten raw in singju, cooked eaten as vegetables or making chutney	October-April	Uncommon
Sita phal IBSD/WEP 041	*Passiflora edulis* Sims	Passifloraceae	Perennial	Climber	Leaf	Cooked eaten as vegetable; added to meat curry	Year round	Common
Torong khongnang IBSD/WEP 042	*Ficus benghalensis* L.	Moraceae	Perennial	Tree	Bud	Cooked eaten by boiling with chilli, dry fish, peas and potato	February-March	Common
Pheija maton IBSD/WEP 043	*Wendlandia glabrata* DC.	Rubiaceae	Perennial	Tree	Inflorescence	Eaten raw as singju or with chutney and also cooked eaten as eromba	December-January	Common
Lamthabi IBSD/WEP 044	*Zehneria scabra* Sond.	Cucurbitaceae	Annual	Climber	Leaf, fruit	Eaten by simply boiling in water with pinch of salt called champhut	July- November	Common
Lamthabi IBSD/WEP 045	*Cyclanthera pedata* (L.) Schrad	Cucurbitaceae	Annual	Climber	Fruit	Eaten raw as snack, cooked eaten as vegetable	November-December	Uncommon
Singjwal IBSD/WEP 046	*Zanthoxylum budrunga* DC	Rutaceae	Perennial	Tree	Leaf	Eaten raw as singju, cooked as vegetable	Year round	Common
Sijou mana IBSD/WEP 047	*Eurya acuminata* DC.	Pentaphylacaceae	Perennial	Tree	Leaf	Cooked eaten in various forms as ooti, chagempomba and eromba	Year round	Common
Ansingteh IBSD/WEP 048	*Lycianthes laevis* (Dunal) Bitter	Solanaceae	Perennial	Herb	Leaf, soft stem	Cooked with meat or boil with rice	October-January	Common
*Anjouteh /Morok maan IBSD/WEP 049	*Solanum nigrum* L.	Solanaceae	Annual	Herb	Leaf	Boil and taken as such; boiled and cooked with rice or meat	November-March	Common
Ookhamen IBSD/WEP 050	*Solanum betaceum* Cav.	Solanaceae	Perennial	Shrub	Fruit	Cooked as vegetable or make Chutney	November-January	Common
*Solunche/Jyan/ Gariyangei IBSD/WEP 051	*Elatostema lineolatum* Wight	Urticaceae	Perennial	Herb	Leaf, tender stem	Cooked eaten by simply boiling or with rice and other vegetables	Year round	Common
*Anpui /BP mana IBSD/WEP 052	*Clerodendrum colebrookianum* Walp	Lamiaceae	Perennial	Shrub	Leaf	Taken by boiling with salt or cooked with other vegetables	Year round	Common
*Tharei sapou/Teravu IBSD/WEP 053	*Piper pedicellatum* DC.	Piperaceae	Perennial	Climber	Leaf	Taken by boiling with pinch of salt; cooked as eromba or with meat	April- May	Common
Pfuchowbu IBSD/WEP 054	*Diplazium esculentum* (Retz.) Sw.	Athyriaceae	Perennial	herb	Leaf	Cooked with daal	November- February	Uncommon
Cholang IBSD/WEP 055	*Allium chinense* G. Don	Amaryllidaceae	1–2 months	Herb	Whole part	Added as spice to dish, eaten raw with chutney	December- February	Common
Huihu IBSD/WEP 056	*Derris wallichii* Prain	Leguminosae	Perennial	Tree	New leaf	Strain boiled water and cooked with potato or as eromba	March-April	Uncommon
*Sinthupi/Galwa IBSD/WEP 057	*Dysoxylum gobara* (Buch.-Ham) Merr.	Meliaceae	Perennial	Tree	Tender stem	Strain boil water and cooked as vegetable	March-May	Uncommon
Chonbe IBSD/WEP 058	*Heteropanax* sp.	Araliaceae	Perennial	Tree	Inflorescence	Cooked as vegetable with dry fish or meat; preparation of chutney	March	Uncommon
Ansah IBSD/WEP 059	*Spilanthes paniculata* Wall. ex DC	Asteraceae	Perennial	Herb	Leaf, inflorescence	Cooked along with other Vegetables	Year round; except summer	Common
Wah-vu IBSD/WEP 060	*Polygonum molle* D. Don	Polygonaceae	Perennial	Herb	Leaf	Cooked eaten as vegetable	March-November	Common
*Pah-vu /Yempat IBSD/WEP 061	*Plantago erosa* Wall	Plantaginaceae	Annual	Herb	Leaf	Cooked as eromba or along with other vegetables	Year round	Common
Pullei manbi IBSD/WEP 062	*Etlingera linguiformis* (Roxb.)R.M.SM	Zingiberaceae	Perennial	Herb	Rhizome	Added as an item in various dish	Year round	Common
Laiwa IBSD/WEP063	*Chimonobambusa callosa (*Munro) Nakia	Poaceae	Perennial	Shrub	New shoot	Boiled and prepare along with chilli, fermented fish, potato and pea	September-December	Common
*Naatwa* IBSD/WEP 064	*Schizostachyum munroi* S. Kumar & P. Singh	Poaceae	Perennial	Shrub	New shoot	Cooked with other vegetables and meat	November- December	Common
Gangru IBSD/WEP 065	*Phrynium placentarium* (Lour.) Merr.	Marantaceae	Perennial	Herb	Rhizome	Boil and taken as such	October - November	Common
Anpuinu IBSD/WEP 066	*Hiptage* sp.	Malpighiaceae	Perennial	Climber	Leaf	Eaten both raw or steam along with chutney	January-June	Common
Moirang khanam IBSD/WEP 067	*Clerodendrum serratum* (L.) Moon	Lamiaceae	Perennial	Shrub	Leaf, inflorescence	Steamed and used for preparation of chutney	August- September	Common
Anthru IBSD/WEP 068	*Momordica dioica* Roxb. ex Willd	Cucurbitaceae	Perennial	Climber	Tender leaf	Cooked by boiling with rice as chagempomba	Year round	Common

*some species have multiple names as they are known by different names in different communities

Further, to perform an integrated assessment of 68 species of wild edible vegetable, the authors used Analytical hierarchy process (AHP) method [36].

### Data analysis

For a systematic approach to integrated assessment, ten evaluation criteria considered important to determine the value of wild edible vegetable were selected, and a score was assigned to each of them (Table 2). These are Taste (T), Distribution (D), Community status (CS), Life form (LF), Basis of civil use (BCU), Wild or cultivated (WC), Edible time (ET), Edible part (EP), Medicinal value (MV), and Market potential (MP).

**Table 2:** Criteria, weight, sub-criteria and assignment score

Assignment criteria	Weight	Sub-criteria	Assignment score
C1- Taste (T)	0.1934	Most preferred	4
		Commonly preferred	3
		Preferred but not common	2
		Occasionally used	1
C2-Distribution (D)	0.1920	7–9 districts	4
		5–6 districts	3
		3–4 districts	2
		1–2 districts	1
C3-Community status (CS)	0.0749	Dominant	3
		Common	2
		Rare	1
C4-Life form (LF)	0.0283	Perennial	2
		Annual/Biennial	1
C5-Basis of civil use (BCU)	0.0576	Wide range	4
		Commonly used	3
		Used but not common	2
		Rarely used	1
C6- Wild or cultivated (WC)	0.0358	Cultivated	2
		Wild	1
C7- Edible time (ET)	0.0933	Cross seasonal eating	2
		Single seasonal eating	1
C8-Edible part (EP)	0.1644	Multiple parts	2
		Single part	1
C9-Medicinal value (MV)	0.0324	Yes	1
		No	0
C10-Market potential (MP)	0.1278	High	3
		General	2
		Low	1

### Weight determination

Weight determination was based on Analytical hierarchy process (AHP) method [36]. According to the relative importance of each evaluation criteria, the weight of each criterion can be determined. This paper applied the subjective weighting method. The weight of each criterion was calculated using the following steps:

- Step1. A hierarchy was constructed based on the ten evaluation criteria viz. Taste (C1), Distribution (C2), Community status (C3), Edible time (C4), Edible part (C5), Life form (C6), Wild or cultivated (C7), Basis of civil use (C8), Medicinal value (C9), and Market potential (C10) with a total of 28 sub-criteria (Table 2).
- Step2. This step is to define the relative importance of each criterion by making a pairwise comparison. The seven-point preference scale of Saaty [37] was used as the fundamental scale for this analysis. If two attributes were equally preferred a score of 1 was assigned, judgement moderately favoured one over other - assignment score 3, one strongly favoured over another - assignment score 5, one very strongly favoured over another - assignment score 7; intermediate values of 2,4,6 were assigned when compromisation needed in decision making. If a criterion was preferred more than the comparison criteria, the reciprocal was assigned to the comparison criteria. The use of reciprocals yields the property that (ai, j)(aj, i) = 1, where ai, j, the preference score of criterion i to criterion j; aj, i, preference score of criterion j to criterion i and ai, j = 1/aj, i [38]. Judgement matrix and consistency check of the evaluation model is constructed in Table 3.

**Table 3:** Judgement of matrix and consistency check of the value criteria

Judgment matrix												Consistency check
	C1	C2	C3	C4	C5	C6	C7	C8	C9	C10	Weight	λ max = 11.1445 CI = 0.1271 RI =1.49 CR = 0.0853 < 0.1
C1	1	2	3	3	2	5	5	3	5	3	0.1934	
C2	1/2	1	3	3	2	3	5	3	5	3	0.1920	
C3	1/3	1/3	1	1/3	1/3	3	3	3	3	1/3	0.0749	
C4	1/3	1/3	3	1	1/3	2	3	3	3	1/3	0.0933	
C5	1/2	1/2	3	3	1	5	5	3	3	3	0.1644	
C6	1/5	1/3	1/3	1/2	1/5	1	1/2	1/3	1/3	1/3	0.0283	
C7	1/5	1/5	1/3	1/3	1/5	2	1	1/3	3	1/5	0.0358	
C8	1/3	1/3	1/3	1/3	1/3	3	3	1	3	1/3	0.0576	
C9	1/5	1/5	1/3	1/3	1/3	3	1/3	1/3	1	1/3	0.0324	
C10	1/3	1/3	3	3	1/3	3	5	3	3	1	0.1275
- Step3. The weights of the decision elements were computed using the eigenvalue (λmax). The consistency index (CI) was computed from the eigenvalue as CI = (λmax- n)/ (n–1). The consistency indices of randomly generated reciprocal matrices from the scale 1to7 are called the random indices, RI. The RI for matrices of order ‘n’ is given in Table 4 [37]. The upper row is the order of the matrix (n), and the lower is the corresponding consistency index of the random judgements. The ratio of ‘CI’ to ‘RI’ for the same order matrix is called the consistency ratio (CR), which defines the accuracy of comparisons. The integrated weight of each of the index and the overall weight is then calculated.

**Table 4:** RI value versus ‘n’

n	1	2	3	4	5	6	7	8	9	10	11	12	13	14	15
RI	0.00	0.00	0.58	0.90	1.12	1.24	1.32	1.41	1.45	1.49	1.51	1.48	1.56	1.57	1.59
- Step4. The integrated value (IV) of each species was calculated using the following formula [39] IV = 0.19348 × T + 0.1920 × D + 0.0749 × CS + 0.0933 × ET + 0.1644 × EP + 0.0283 × LF+ 0.0358 × WC + 0.057 × BCU + 0.0324 × MV + 0.1275 × MP.

## Results and discussion

### Main characteristics and consumption pattern of wild edible plants

**Fig. 2:**
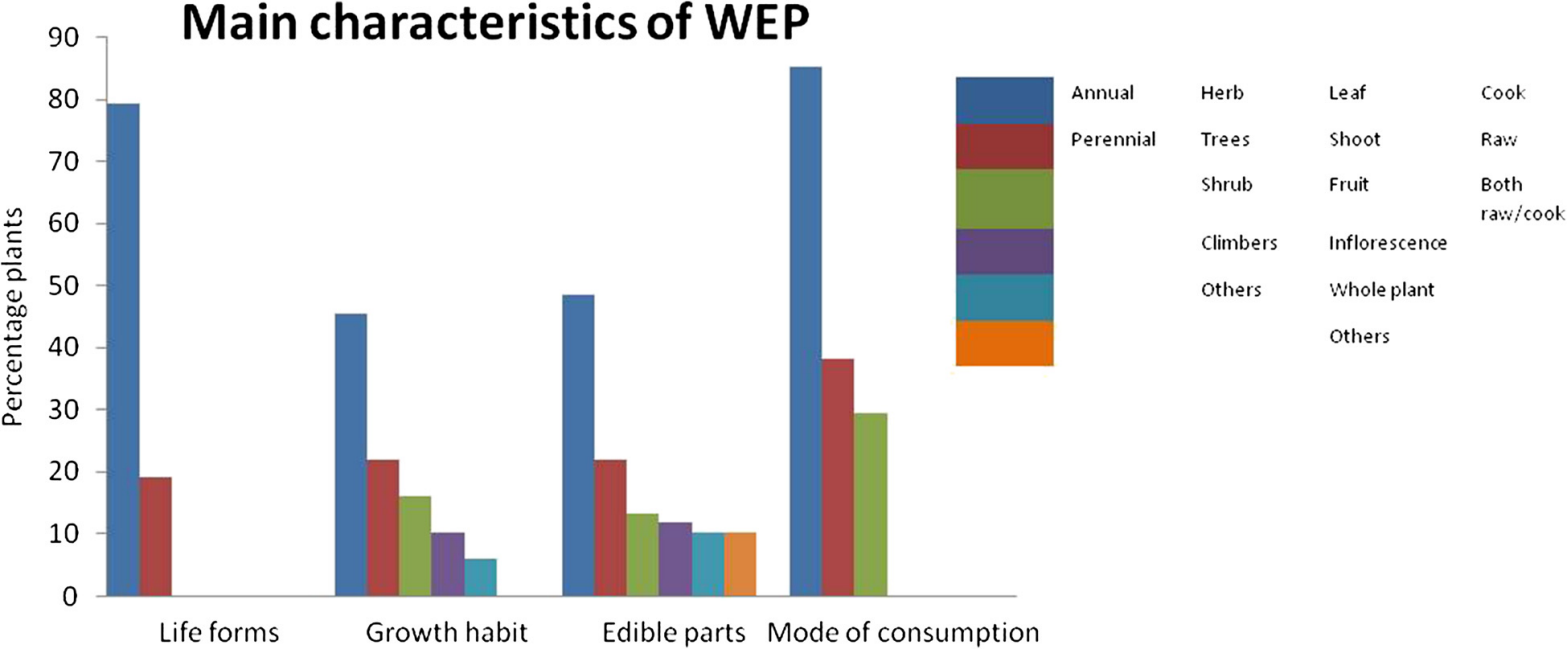
Main characteristics showing life forms, Growth habit, edible parts and mode of consumption of WEPs

The northeast region of India, a major part of the Indo-Burma hotspot, supports considerable biodiversity. The people of the state are traditionally dependent on the wild plant resources for various cultural and religious purposes since ancient times [39]. A large variety of such edible plants are also sold in the market as a means of livelihood for the rural population. This study highlighted the rich floral diversity and the traditional knowledge of the use of wild plants as a source of vegetable by the ethnic communities of Manipur. A total of 68 wild species belonging to 42 families have been documented and collected from the market survey. The list of plants along with their local name, life form, growth habit, use category, collection period, parts consumed, mode of consumption, availability status are presented (Table 1). Of these species, 54 (79 %) are perennial while others are annual (19 %).Their growth habit includes tree, shrub, herb, climber, creeper, weed and hydrophytes. Herbaceous plants make up the highest proportion of edible plants with 31 species (46 %), followed by trees with 15 species (22 %) and shrubs with 11 species (16 %). Among the edible parts, leaves are dominant with 33 species (49 %) followed by shoot and stem with 15 species (22 %), and most of them are consumed as cooked vegetables that include boil, steam, and fry (Fig. 2). Further, 57 species (84 %) are commonly available whereas 11 (16 %) are rare. As many as 51 species (75 %) are seasonal, and 17 (25 %) are available throughout the year. Some of them are used as herbal medicine while others are used as poultry feed, fuelwood, fencing, etc. besides their use as food. The multipurpose use of these plants can be an important reason for their conservation [40].

**Fig. 3:**
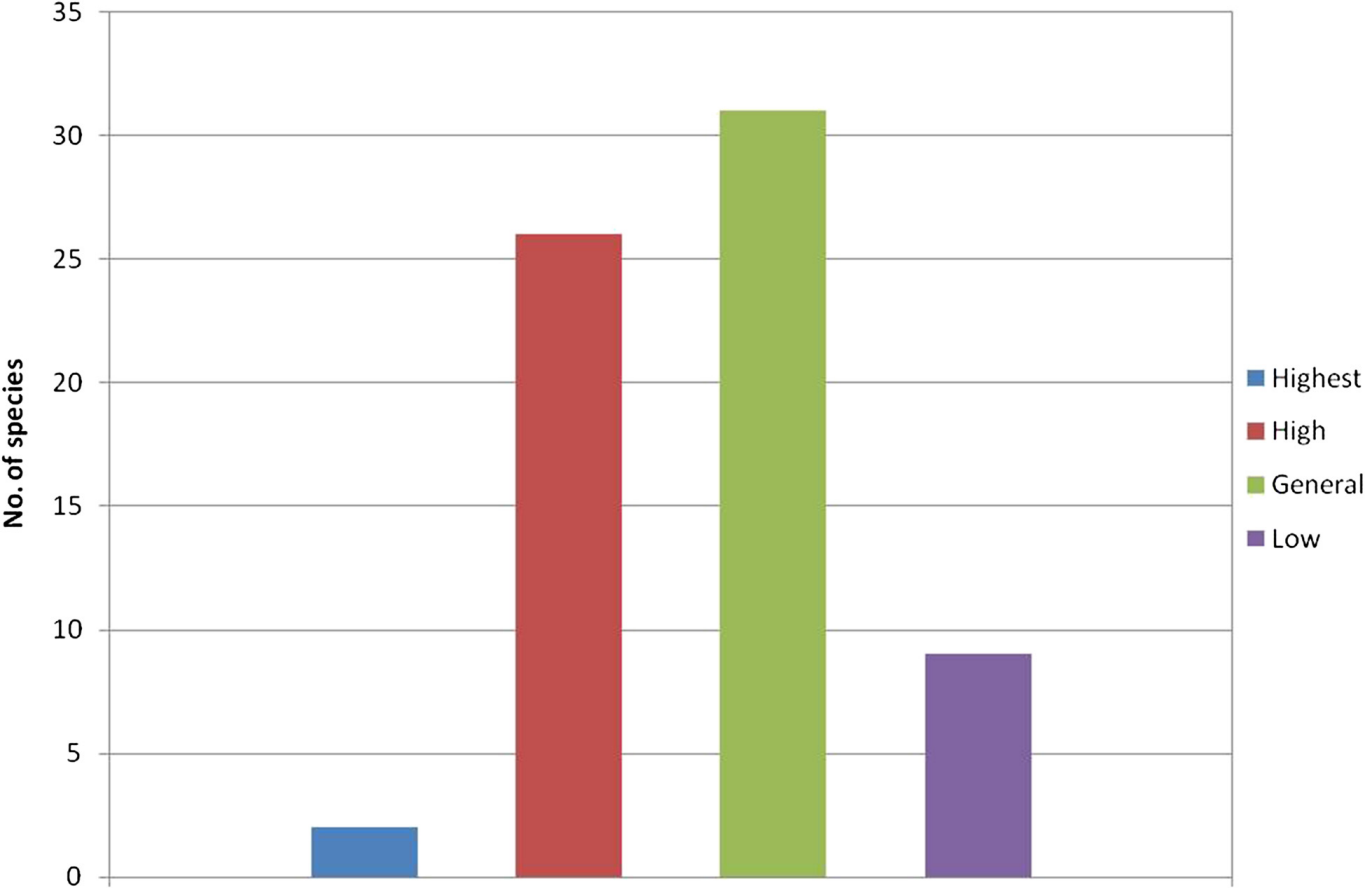
Categories of the Integrated values of wild edible vegetables

The communities use different modes of consumption of these species. Though the method of preparation is same, use of wild vegetables varies among communities according to their preference in taste and food habit. Singju, Eromba, Ooti, Chagempomba, Kangsoi, Champhut are the major traditional cuisines unique to the state that form an important part of daily diet. Use of one or more wild vegetables is a necessary part of a local meal. Fresh collection of vegetables before cooking is preferred.

The description of the mode of preparation of traditional dishes (Table 1) is as follows: (i) Singju, the raw dish is most common traditional food (traditional salad) made by mixing a number of wild edible species with fermented fish, chilli, and other plants (like cabbage); (ii) Eronba is prepared by boiling plant parts and smashing it with potatoes or pea, chilli, and fermented fish into a semi-liquid dish; (iii) Ootti is prepared by boiling vegetable mixture along with some rice with a pinch of sodium bicarbonate; (iv) Chagempomba is prepared by boiling soyabean, rice and different varieties of vegetables and (v) Kangsoi is a soupy dish prepared by boiling vegetable and potato with chilli, salt, fermented fish and small dried fish (see Additional file 1). Such use of WEPs in traditional delicacies was common among the tribal communities in the Himalayan Mountains [41] which explains their role in diversifying diet and fulfilling the nutritional requirement of the local system.

### Analysis of evaluation criteria

#### Taste, market potential and medicinal value

Previous ethnobotanical surveys indicated that organoleptic traits can be used as the basis for value judgement and became criteria against which the value for a range of species could be evaluated [42]. For e.g. when the respondents are asked to choose which of the given two species is more significant or valuable to them, the response for one species being more valuable or significant than other is simply because it is tastier than the other. The survey participants indicated that the tastiest species are most commonly preferred by consumers, and have greater market potential though marketability is also influenced by other factors such as abundance, availability, distribution. Taste was the most important criterion for preference in case of leafy vegetables in southern Ethiopia [17], and also in Benin [43]. The taste criterion is based on the method of Jain et al. [30]. Some of the plants though not considered tasty are consumed by the locals for their medicinal quality or the health benefit they provided. Based on the survey data and literature review, 44 species (65 %) in the present study are with ethnomedicinal property. According to a report on wild vegetable resources in Inner Mongolia, 62 species of wild vegetable are used as medicine [38].

#### Distribution, community status and life form

The majority of the wild plants are distributed in most of the region whereas the rest are found in a certain area. As for the community status of the 68 species surveyed, 51 species (75 %) are common, 11 species (16 %) are rare, and 6 species (9 %) make up the dominant species. Of these species, 54 are perennial (79 %) while the rest are annual (19 %). These conditions directly influence the collection and consumption of these species.

#### Frequency of use and whether the plant is wild or domesticated

Based on the survey, 22 species (32 %) are widely used for frequent consumption, 42 species (61 %) are commonly used while 4 species (6 %) are rarely used. Some of the most widely used species are *Euryale ferox, Chimonobambusa callosa, Ipomoea aquatica, Oenanthe javanica, Alocasia cucullata, Neptunia oleracea, Houttuynia cordata, Hedychium coronarium*, *Alpinia nigra, Amomum aromaticum, Eryngium foetidum, Passiflora edulis, Ficus benghalensis, Zanthoxylum budranga*. It could be attributed to their taste, availability in multiple seasons or high abundance in its season and their use in various cuisines. Of the 68 species, 31 species (46 %) are semi-domesticated. They have been put into cultivation practice especially in kitchen garden while the remaining species are found in the wild. It is observed that people focus on planting those species that have good market value, taste and consumed more frequently. Usually, the tastiest species also score high concerning marketability (based on survey). Kidane et al. [17] emphasized the importance of home gardens for promotion and cultivation of prioritized leafy vegetables for ease of management, ownership, supervision and intensive cultural practices in cultivated land.

#### Edible parts and edible time

Sixteen species (24 %) are consumed for more than one part of the plant whereas 52 species (76 %) are collected for their single part. The edible parts include leaf, stem, fruit, root, rhizome, bud, tuber and inflorescence. Among them, leaves are dominant followed by shoot and stem and most of these are consumed as cooked vegetables. Consumption of 40 species (59 %) is single seasonal, and 28 species (41 %) are used in multiple seasons.

#### Integrated value

According to the integrated value (Table 5), the wild vegetables in Manipur can be classified into 4 categories (Fig. 3) – highest (integrated value > 2.5), high (integrated value 2.0 - 2.5), general (integrated value1.5 - 2.0) and low (integrated value <1.5). There are only 2 species with the highest value, 26 species with high value, 31 species with general value and 9 species with low value. Overall, 57 species (84 %) have a high or general value. Some high scoring vegetables include *Centella asiatica, Euryale ferox, Chimonobambusa callosa, Ipomoea aquatica, Alocasia cucullata, Neptunia oleracea, Hedychium coronarium, Eryngium foetidum, Ficus benghalensis, Cycus pectinata, Cissus javanica, Wendlandia glabrata,* and *Elatostema lineolatum.* It could be due to their traits of high-quality vegetables such as unique taste, appropriate edible parts, high abundance in its season, ease of processing, high market value and so on. They are also among the most preferred and frequently consumed species.

**Table 5:** Integrated values of evaluation criteria of the wild edible vegetables of Manipur

Local names	Scientific names	T	D	CS	LF	BCU	WC	ET	EP	MV	MP	IV
Chuchurangmei	*Sesbania sesban* (L.) Merr.	3	2	2	1	2	1	1	2	1	1	1.87
Kolamni	*Ipomoea aquatica* Forssk.	3	2	3	2	3	1	2	1	1	2	2.09
Komprek	*Oenanthe javanica* (Blume) DC.	3	3	2	2	3	1	2	1	1	1	2.08
Sinjupaal	*Alocasia cucullata* (Lour.) G. Don	4	2	3	2	3	2	2	1	0	3	2.41
Yelang	*Polygonum barbatum* L.	3	2	2	1	2	2	1	1	1	1	1.74
Kakthum	*Eleocharis dulcis* (Burm. f.) Trin. ex Hensch	3	2	2	1	1	1	1	1	0	2	1.74
Ching yensil	*Antidesma diandrum* (Roxb.)B. Heyne. ex Roth	3	2	2	2	2	1	2	1	1	2	1.96
Kengoi	*Lysimachia ovovata* Buch.-Ham. ex Wall	3	2	2	1	2	2	1	1	1	1	1.74
Peruk	*Centella asiatica* (L.) Urb.	4	3	3	2	4	2	2	2	1	2	2.73
Thambou	*Nelumbo nucifera* Gaertn.	3	2	2	2	2	1	2	2	1	3	2.25
Tharo	*Nympheae nouchali* Burm. f.	2	2	2	2	1	1	1	2	1	1	1.75
Thangjing	*Euryale ferox* Salisb.	4	2	2	1	4	2	1	1	1	3	2.30
Esing ekaithabi	*Neptunia oleracea* Lour.	4	2	2	1	3	2	1	1	1	3	2.25
Koukha	*Sagittaria sagittifolia* L.	3	2	2	1	2	1	1	1	1	2	1.83
Yendang	*Cycas pectinata* Buch.-Ham	4	2	1	2	2	1	1	1	1	3	2.11
Monsaobi	*Chenopodium album* L.	2	2	2	1	2	1	1	1	1	1	1.51
Kanghumaan	*Meriandra bengalensis* (Roxb.) Benth.	1	2	1	2	1	2	2	1	1	2	1.47
Tekta	*Pogostemon purpurascens* Dalzell	3	2	1	1	2	2	1	1	0	2	1.76
Yerum keirum	*Stellaria media* (L.) Vill.	3	2	2	1	2	1	1	2	1	1	1.87
Toninkhok	*Houttuynia cordata* Thunb.	3	3	3	2	3	2	2	2	1	2	2.48
Loklei	*Hedychium coronarium* J. Koenig	4	3	2	2	3	2	1	1	1	3	2.47
Pullei	*Alpinia nigra* (Gaertn.) Burtt	3	3	2	2	3	2	1	1	1	2	2.15
Namra	*Amomum aromaticum* Roxb.	3	3	2	2	3	2	1	1	1	2	2.15
Yaipal	*Curcuma angustifolia* Roxb.	2	2	2	2	2	2	1	1	1	2	1.7
Sarei mapan	*Amomum* sp.	3	2	1	2	2	1	1	1	0	2	1.76
Esing kambong	*Zizania latifolia* (Griseb.) Turcz. ex Stapf	4	2	1	2	2	1	1	1	1	3	2.11
Chantruk mana	*Cardamine hirsute* L.	2	2	2	1	2	2	2	1	1	2	1.76
Huikhong/ Mansam	*Viola pilosa* Blume	3	2	2	2	2	1	2	2	1	2	2.12
Phunil	*Gnaphalium indicum* L.	2	2	2	1	2	2	1	2	1	2	1.83
Kongouyen	*Cissus javanica* DC	3	3	2	2	2	1	2	1	1	2	2.12
Heibi mana	*Vangueria spinosa* (Roxb. ex Link) Roxb.	2	2	2	2	2	2	1	1	0	2	1.64
Lam khamen	*Solanum torvum* Sw.	1	2	2	2	2	1	1	1	0	1	1.32
Nongmangkha	*Phlogacanthus thyrsiformis* (Roxb.ex Hardw.) Mabb	3	3	2	2	2	2	1	2	1	2	2.25
Oosingsha mapaan	*Litsea cubeba* Pers.	2	3	2	2	2	1	1	2	1	2	2.02
Chigonglei angouba	*Leucaena leucocephala(*Lam.) de Wit	2	2	2	2	2	2	1	1	1	2	1.9
Oothum maton	*Wendlandia paniculata* (Roxb.) DC.	2	3	2	2	2	2	1	1	0	2	1.67
Mukthrubi	*Zanthoxylum acanthopodium* DC.	3	3	2	2	2	2	2	1	1	2	2.18
Naoseknambi / Anpajul	*Zanthoxylum* sp.	3	1	2	2	2	2	1	1	0	2	1.67
Awaphadigom	*Eryngium foetidum* L.	4	3	2	2	3	2	2	1	1	3	2.56
Heiba mana	*Exbucklandia populnea* (R.Br. ex. Griff.) R.W. Br.	3	2	2	2	2	2	2	1	0	2	1.96
Sitaphal mana	*Passiflora edulis* Sims	3	3	2	2	3	2	2	2	1	2	2.41
Torong khongnang	*Ficus benghalensis* L.	4	3	2	2	3	1	1	2	0	2	2.43
Pheija mapan	*Wendlandia glabrata* DC.	3	3	2	2	3	1	1	1	1	2	2.11
Lamthabi mana	*Zehneria scabra* Sond.	3	1	1	2	2	1	1	1	0	2	1.56
Lamthabi	*Cyclanthera pedata* (L.) Schrad.	2	1	1	2	2	1	1	2	1	3	1.69
Singjwal	*Zanthoxylum budrunga* DC	4	1	3	2	3	1	2	1	0	2	2.05
Sijou mana	*Eurya acuminata* DC.	3	1	3	2	3	2	2	1	0	1	1.77
Ansingteh	*Lycianthes laevis* (Dunal) Bitter	2	1	2	2	2	1	1	1	0	2	1.44
Anjouteh/Morokmaan	*Solanum nigrum* L.	2	3	2	1	2	1	2	1	1	2	1.92
Ookhamen	*Solanum betaceum* Cav.	2	2	1	2	2	2	2	1	1	2	1.72
Solunche/Jyan/Gariyangei	*Elatostema lineolatum* Wight	4	2	2	2	3	1	2	2	0	2	2.34
Anpui/Bp mana	*Clerodendrum colebrookianum* Walp	3	2	2	2	3	1	2	1	1	2	2.01
Tharei sapou/Teravu/Thimnahan	*Piper pedicellatum* C. DC.	3	1	2	2	3	1	2	1	1	2	1.81
Pfuchowbu	*Diplazium esculentum* (Retz.) Sw	2	1	2	2	2	2	1	1	0	2	1.48
Cholang	*Allium chinense* G. Don	2	2	2	1	2	2	2	2	1	2	1.93
Huihu	*Derris wallichii* Prain	3	1	1	2	2	1	2	1	0	2	1.66
Sinthupi/Galwa	*Dysoxylum gobara* (Buch.-Ham) Merr	2	1	1	2	2	1	1	1	0	3	1.49
Chonbe	*Heteropanax* sp.	3	1	2	2	3	2	1	1	0	3	1.86
Ansah	*Spilanthes paniculata* Wall. ex DC	2	3	2	2	2	1	2	2	1	2	2.12
Wah-vu	*Polygonum molle* D. Don	2	1	2	2	2	1	1	1	0	2	1.44
Pah-vu/yempat	*Plantago erosa* Wall	3	3	2	2	3	1	1	1	1	2	2.11
Pullei manbi	*Etlingera linguiformis* (Roxb.) R.M. Sm.	3	2	2	2	2	2	2	1	1	2	1.99
Laiwa	*Chimonobambusa callosa* (Munro) Nakia	4	2	2	1	4	1	1	1	0	3	2.24
Naatwa	*Schizostachyum munroi*	2	2	2	2	1	1	1	1	0	2	1.58
Gangru	*Phrynium placentarium* (Lour.) Merr.	3	1	1	2	2	2	1	1	0	2	1.6
Anpuinu	*Hiptage* sp.	2	1	2	2	2	1	1	1	1	1	1.35
Moirang khanam	*Clerodendrum serratum* (L.) Moon	1	2	2	2	2	1	2	1	1	1	1.44
Anthru	*Momordica dioica* Roxb.ex Willd.	2	1	2	2	2	1	2	1	0	1	1.41

An integrated assessment of wild species has not yet been done in Manipur and elsewhere except Inner Mongolia, China [38]. It provides scientific clues to select priority and high-quality species. The present study developed a new approach to the integrated assessment of wild leafy vegetables based on a set of defined criteria. The result highlighted that 57 species (84 %) have good to high value (Table 5). Among the high scoring species, *Zanthoxylum budrunga, Passiflora edulis, Clerodendrum colebrookianum, Spilanthes paniculata, Cissus javanica, Elatostema lineolatum, Plantago erosa, Litsea cubeba,* etc. and other species such as *Zehneria scabra, Cyclanthera pedata, Piper pedicellatum, Solanum nigrum, Eurya acuminate, Solanum betaceum, Allium chinense, Heteropanax* sp*., Dysoxylum gobara, Diplanzium esculantum, Etlingera linguiformis, Derris wallichii, Phrynium placentarium* are found to be consumed mainly by the tribal communities and rarely known to other communities. It may be due to their traditional food habit experience, preference, and local species availability. Many more such unexplored leafy vegetables are believed to exist. There is a need for exploitation of such unexplored resources given the storehouse of traditional knowledge the tribal possessed. It will provide a way for screening newer and alternative source of nutrition.

The present finding will be useful in the evaluation of nutritional components of high priority species for their integration into the agricultural system based on nutritive values. Further, assessing their cultivable potential and working towards developing agro-techniques can bring more potential species under domestication for conservation through sustainable use. Moreover, it will also help to understand their role in future food and nutritional security of the state.Therefore, documentation and prioritization would ensure that the highest priority species is preserved for use in crop improvement programs and contribute towards achieving the goal of food and nutritional security.

#### Traditional knowledge (TK)

WEPs constitute an integral part of the indigenous socio-ecological system associated with traditional ecological knowledge of ethnic communities. We observed that women (>40 years old) of a household possessed more traditional knowledge about leafy vegetables including the identity of the species, usage, and mode of preparation. It could be due to their association with household chores such as cooking, marketing, and their home nurturing qualities. Upetry et al. [18] have reported a similar finding. Phillips and Gentry [44] also reported that WEP knowledge is gained early in life and increases with age.

Participants in the survey have mentioned a declined in the traditional knowledge of natural resources in recent times. The cultural and traditional association of WEPs with the ethnic communities is gradually falling as they are not passed down to future generations, so present generations have little information regarding wild edibles associated with the diet of their ancestors. These generations are no longer interested in acquiring traditional knowledge of WEPs. Presumably, increasing level of modernization significantly contributes to the erosion of TK. Benz et al. [45] explained the abandoning of aboriginal ancestral practices by indigenous people in Mexico for economic and social gain. Loss of knowledge may occur if resources disappear from the landscape. Srivastava and Singh [46] have reported that frequent and overexploitation of species leads to threat in survival for some species of Northeastern States. However, the loss of indigenous knowledge worldwide has varied reasons and has been explained under local, ecological, socio-economic and cultural contexts [17]. Studying major grounds for the decline of resources and loss of associated knowledge will help decision makers in their formulations and analysis of policy [47]. Documentation and evaluation of traditional knowledge related to diversity, usage, and status of WEPs are crucial for preserving it for future generations. Support of TK systems and empowerment of its knowledge holders, promotion of the use of TK, recognition of rights of TK holders relating to their knowledge are the bottom-up approach to development [48]. It should be supported by complementary in-situ an ex-situ conservation strategies to conserve and sustainably utilize the natural resources and associated knowledge.

## Conclusion

The diverse use of wild plant resources for food, medicine, income and socio-cultural purposes by the ethnic communities of Manipur revealed the high dependence on them with as many as 68 wild vegetables documented and collected. Though Manipur is bountiful in wild vegetable, a large number of them remain unexplored or known to certain sections of society. Traits that contribute to the uncommon usage of these plants include different food habits and experience of ethnic communities, the difference in taste preference, distribution, abundance and edibility time.

According to the integrated assessment, 57 out of 68 (84 %) species have good to high value. These high scoring species exhibit the traits of high-quality vegetables, such as taste, appropriate edible parts, multiple edible parts, availability, abundance, easily cultivable, simple to collect and process, and so on. To increase dietary diversity and livelihood sustenance of local people, complimentary studies and further ethnobotanical studies will be conducted. The traditional knowledge and understanding of wild food plants may serve as baseline data for future research and development activities and further biotechnological intervention. A detailed evaluation of nutritional components of the potential species should be conducted for integration into the agricultural system based on their nutritive values and for the conservation of elite germplasm. Further studies should also be done to assess their cultivable potential and work towards developing propagation and agro-techniques to bring more potential wild species under domestication for sustainable utilization of natural resources. Furthermore, proper value chain development for marketing and value-addition of selected species can facilitate enough income to native communities. Documentation and conservation of highest priority species would ensure they are available for use in genetic improvements of crop species as a contribution towards food and nutritional security. Therefore, communities should engage in sustainable management and preservation of traditional knowledge of these multi-valued resources for the well-being local communities.

## Additional file

Additional file 1:
**Major cuisines of Manipur.** (PDF 481 kb)
