# Erratum to: Assessment of wild leafy vegetables traditionally consumed by the ethnic communities of Manipur, northeast India

**DOI:** 10.1186/s13002-016-0083-1

**Published:** 2016-02-08

**Authors:** Surjata Konsam, Biseshwori Thongam, Arun Kumar Handique

**Affiliations:** Plant Systematics and Conservation Laboratory (PSCL), Institute of Bioresources and Sustainable Development (IBSD), Takyelpat, Imphal, 795001 Manipur India; Department of Biotechnology, Gauhati University, Guwahati, 781014 India

Erratum:

The original version of this article [1] was published with an incorrect author order. The author order should read Surjata Konsam, Biseshwori Thongam, and Arun Kumar Handique. The original article has also been updated to present the author list in its correct order.
