# Supplementary material for: Assessment of wild leafy vegetables traditionally consumed by the ethnic communities of Manipur, northeast India
Source: J Ethnobiol Ethnomed. 2016 Jan 29;12:9. doi: 10.1186/s13002-016-0080-4 (PMC4731935; doi:10.1186/s13002-016-0080-4)
Supplement: Additional file 1: — Major cuisines of Manipur. (PDF 481 kb) [file 13002_2016_80_MOESM1_ESM.pdf]

Major traditional cuisines of Manipur:

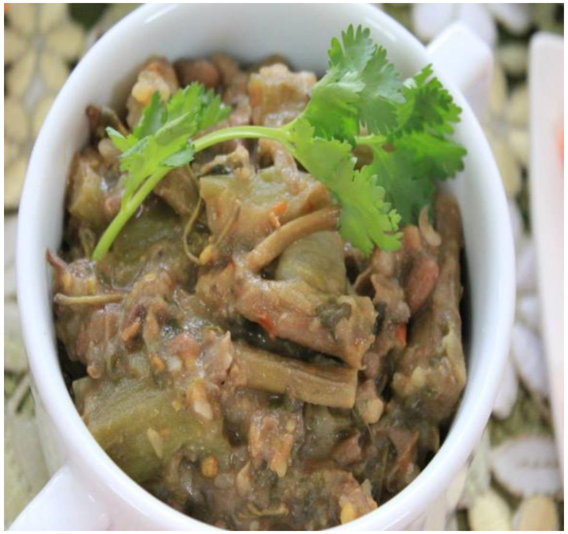
Eromba

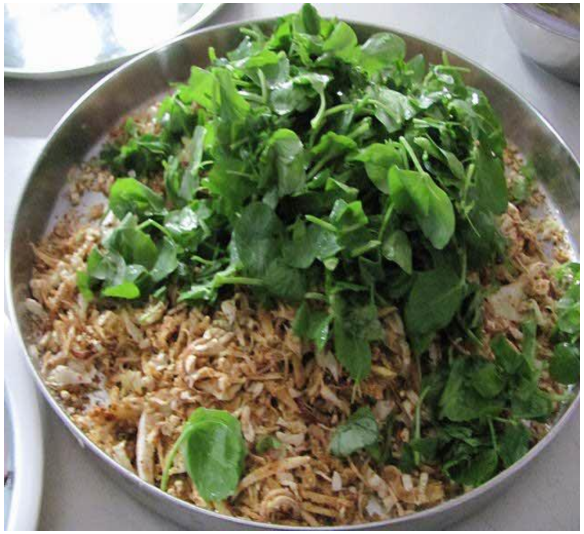
Singju

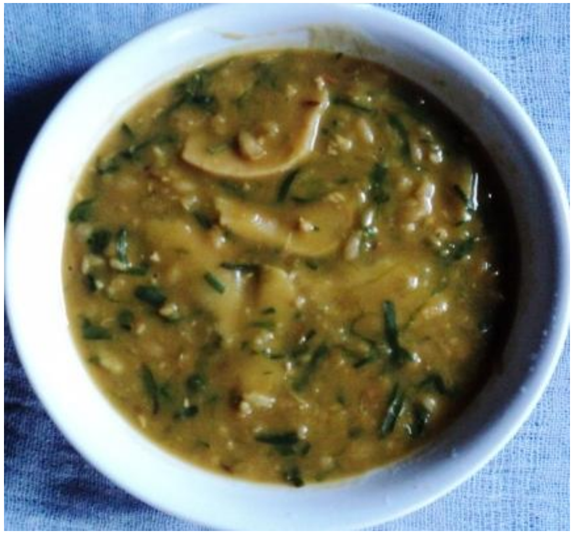
Ooti

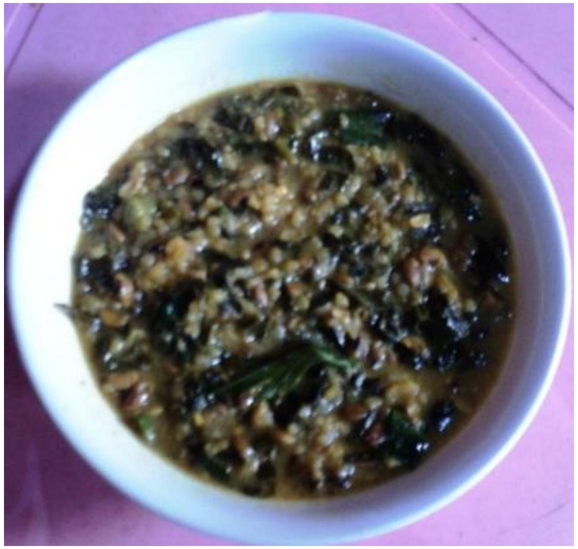
Chagempomba

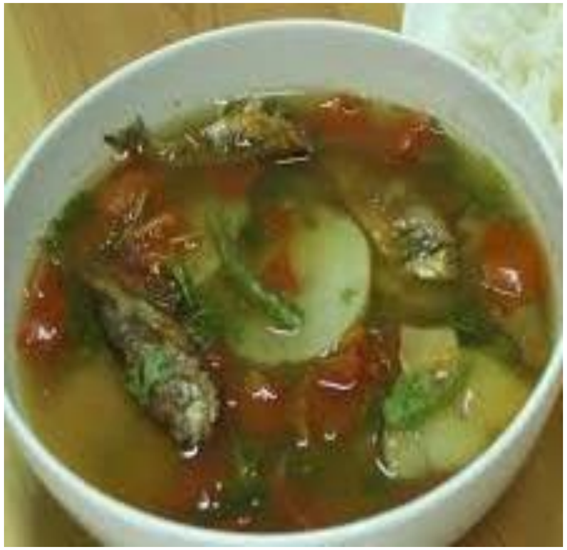
Kangsoi

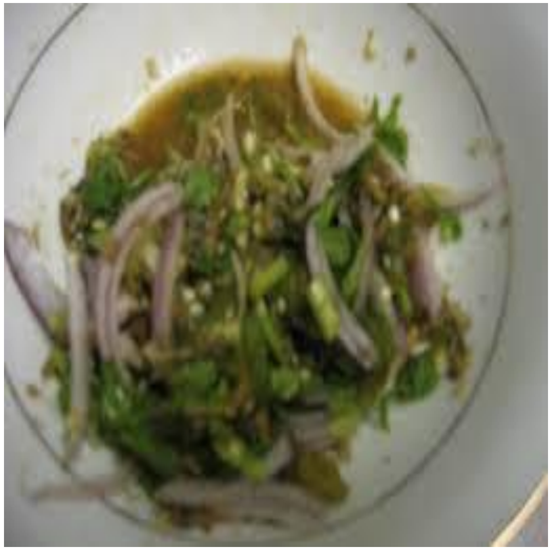
Ametpa

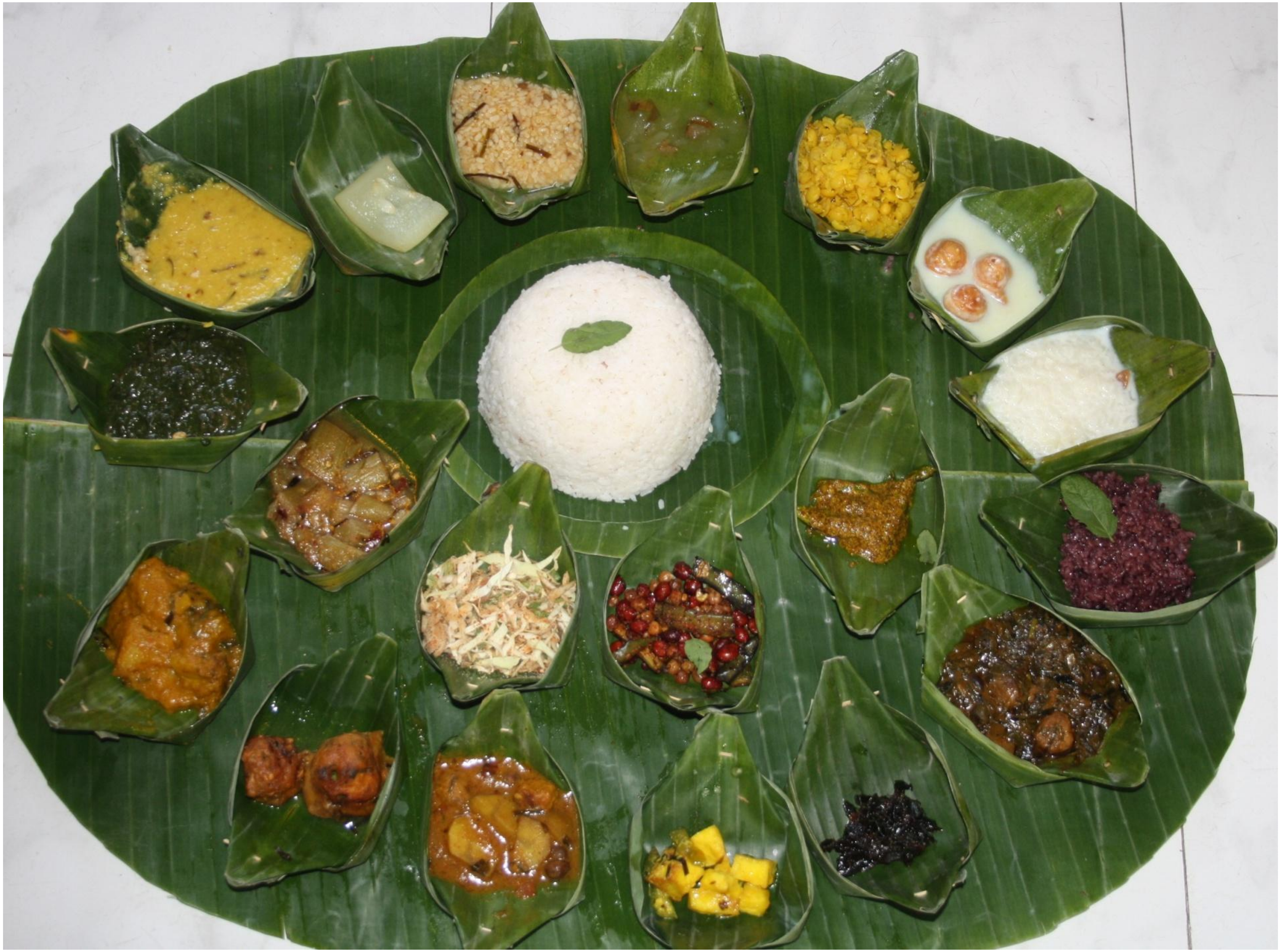

A traditional feast being served on religious ceremony.
